# Retinoic Acid Signaling Regulates the Metamorphosis of Feather Stars (Crinoidea, Echinodermata): Insight into the Evolution of the Animal Life Cycle

**DOI:** 10.3390/biom10010037

**Published:** 2019-12-25

**Authors:** Shumpei Yamakawa, Yoshiaki Morino, Hisanori Kohtsuka, Hiroshi Wada

**Affiliations:** 1 Graduate School of Life and Environmental Sciences, University of Tsukuba, 1-1-1 Tennodai, Tsukuba, Ibaraki 305-8572, Japan; 2 Misaki Marine Biological Station, School of Science, University of Tokyo, 1024, Misaki, Miura, Kanagawa 238-0225, Japan

**Keywords:** retinoic acid signaling, metamorphosis, feather stars, echinoderms, evolution of life cycle

## Abstract

Many marine invertebrates have a life cycle with planktonic larvae, although the evolution of this type of life cycle remains enigmatic. We recently proposed that the regulatory mechanism of life cycle transition is conserved between jellyfish (Cnidaria) and starfish (Echinoderm); retinoic acid (RA) signaling regulates strobilation and metamorphosis, respectively. However, the function of RA signaling in other animal groups is poorly understood in this context. Here, to determine the ancestral function of RA signaling in echinoderms, we investigated the role of RA signaling during the metamorphosis of the feather star, *Antedon serrata* (Crinoidea, Echinodermata). Although feather stars have different larval forms from starfish, we found that exogenous RA treatment on doliolaria larvae induced metamorphosis, like in starfish. Furthermore, blocking RA synthesis or binding to the RA receptor suppressed metamorphosis. These results suggested that RA signaling functions as a regulator of metamorphosis in the ancestor of echinoderms. Our data provides insight into the evolution of the animal life cycle from the viewpoint of RA signaling.

## 1. Introduction

The life cycle of many marine invertebrates includes a shift from swimming as a planktonic larva with cilia to a benthic adult [1]. Various larval forms exist in animals, including sponges, cnidarians, and various bilaterians; this has attracted the interest of many zoologists to the origin of the larvae and evolution of the life cycle [1,2,3]. The patterning mechanism of the larval body is conserved in various animal groups, including Protostomes, Deuterostomes, and Cnidaria, suggesting an older evolutionary origin of planktonic larvae [4,5,6,7]. Nevertheless, as Raff [8] hypothesized that larval forms evolved multiple times over the course of evolution, the evolution of the life cycle in the animal kingdom is still controversial. Therefore, in addition to the morphological aspects, it is important to understand the evolution of the regulatory mechanisms underlying the life cycles of marine invertebrates.

The life cycle transition in jellyfish (Cnidaria) and starfish (Echinoderm) is regulated by the conserved machinery of retinoic acid (RA) signaling [9,10]. Planktonic larvae of many marine invertebrates settle on an external substrate (settlement) and subsequently transit to a benthic adult phase (metamorphosis) [2]. In jellyfish, the planula larvae settle on the seafloor and commence the polyp stage; subsequently, environmental signals, including cold temperatures, stimulate strobilation and the transition to ephyra stage [9]. Fuchs et al. [9] suggested that endogenous RA mediates the regulation of strobilation after environmental signals are received. On the other hand, when the competent starfish larvae settle on the external substrate using brachiolar arms, they transition to the juvenile stage through metamorphic processes such as enlargement of the juvenile rudiment [10,11]. Yamakawa et al. [10] suggested that, like in jellyfish, RA signaling mediates the regulation of metamorphosis in starfish larvae after environmental cues are received [10]. Although different types of receptors for RA are used in each lineage [9,10], these findings suggest that the RA functions widely in the life cycles of marine invertebrates. To demonstrate this idea, it is necessary to clarify the function of RA signaling in various animal groups. Notably, RA signaling might not function in the metamorphosis of marine annelids. Handberg-Thorsager et al. [12] showed that RA receptor functions as a low-affinity sensor triggering neural differentiation but did not report a metamorphosis-regulating function in a study of trochophore and early nectochaete larvae.

In the present study, we made an attempt to determine the ancestral function of RA signaling in echinoderms. Echinoderms comprise five classes: the most basal Crinoidea and their sister group, Eleutherozoa, consisting of Echinozoa (Echinoid and Holothuria) and Asterozoa (Asteroid and Ophiuroid) [13]. Notably, the larval morphology and the machinery for settlement vary among echinoderm taxa [14,15,16,17]; for example, planktotrophic pluteus larvae of sea urchins and brittle stars settle to the sea bottom using tube feet, while in crinoids, lecithotrophic doliolaria larvae settle using adhesive tufts. Furthermore, it should be noted that the regulation of metamorphosis in sea urchins has been clarified in relatively great detail [18,19,20]; thyroid hormone and histamine signaling modulate larval growth and the acquisition of competency. Although previous studies have suggested that nitric oxide signaling negatively controls the post-settlement process and that the receipt of environmental cues decreases nitric oxide synthesis to commence metamorphosis [21,22], it has not been reported that RA signaling is involved in the regulation of metamorphosis in sea urchins. Therefore, it is unclear whether metamorphosis in echinoderm ancestors is regulated by RA signaling as in starfish.

Here, we investigated whether RA signaling regulates metamorphosis in the feather star (Crinoidea), *Antedon serrata*. We treated doliolaria larvae of *A. serrata* with exogenous RA, resulting in the induction of cystidean larvae. In contrast, metamorphosis was suppressed by treatment with RA synthesis inhibitor and antagonist for RA receptors. In conclusion, our study suggests that RA signaling functions as a regulator of metamorphosis in the ancestor of echinoderms, providing insight into the evolution of the animal life cycle from the viewpoint of RA signaling.

## 2. Materials and Methods

### 2.1. Sampling and Culture of Larvae

We collected adult specimens of *A. serrata* with fertilized eggs or embryos in their pinnular surface from Misaki (Miura, Kanagawa Prefecture, Japan) and Onahama (Iwaki, Fukushima Prefecture, Japan). We incubated the adult specimens in artificial sea water at 14 °C. For experiments, we used doliolaria larvae that hatched from the pinnular surface of adults.

### 2.2. Immunohistochemistry

We fixed the larvae in 4% PFA in MOPS buffer and washed them with phosphate-buffered saline (PBS) with 0.1% Tween 20 buffer (PBST). The fixed embryos were then labeled with anti-acetylated tubulin antibody (Sigma, St. Louis, MO, USA) in a solution containing 0.5% blocking reagent (Roche, Basel, Switzerland), followed by Alexa Fluor 555 goat anti-mouse IgG antibody (Thermo Fisher Scientific, Waltham, MA, USA). Stained embryos were washed with PBST and then observed under a fluorescence microscope.

### 2.3. Reagent Treatments

We prepared 100 mM stock of all-trans RA (Sigma-Aldrich, St Louis, CAS number: 302-79-4), 1 M stock of N, N-diethylaminobenzaldehyde (DEAB, Tokyo Chemical Industry, Tokyo, Japan, CAS number: 120-21-8) and 50 mM stock of RO41-5253 (RO, Focus Biomolecules, Plymouth Meeting, PA, USA, CAS number: 144092-31-9) in dimethyl sulfoxide (DMSO). We incubated the larvae in 2 mL of artificial seawater containing 2 µL of reagents or DMSO in 12-well plates at 14 °C. For the experiment without a substrate, 10 larvae were incubated in one well. Natural sands from Misaki (Miura, Kanagawa Prefecture, Japan) were used for the experiments conducted to induce metamorphosis. In these experiments, a single larva was cultured in one well to identify individuals. For cases in which reagent treatment continued for more than two days, we changed the seawater with the same concentration of reagents every other day.

We evaluated the attachment of larvae to the external substrates by an adhesive tuft as settlement and judged whether the larvae were metamorphosed by clear formation of the calyx, stalk and adhesive plate. From these observations, the numbers of individuals who settled and metamorphosed were counted. The rates of settlement and metamorphosis were calculated by dividing by the number of treated larvae and the number of settled larvae. We carried out experiments using two batches of larvae hatched from different adults. In particular, experiments were conducted once to several times in each batch.

### 2.4. Statistical Analysis

As in our previous work [10], we examined the statistical analyses to evaluate differences in the effects of the treatments of substrate or reagents on settlement or metamorphosis. We used analysis of variance (ANOVA) and the R statistical software package [23]. In our analyses, the conditions and batches described above were assumed to be main factors and “blocks,” respectively. Note that preliminary testing of some treatments revealed slight, but not essential, differences between batches.

### 2.5. Construction of the Phylogenic Trees

We obtained RA signaling-related genes (*raldh*, *rar*, and *rxr*) from the transcriptome data assembled previously for the crinoid *Metacrinus rotundus* [24]. In addition to the previously used datasets [10], a sequence alignment was performed using MAFFT (default value in the online version) and the phylogenetic sequence was filtered using trimAL with a gap threshold of 0.8 [25,26]. The estimate of the amino acid substitution model and preparation of the maximum likelihood tree were carried out using RAxML [27]. Confidence values were calculated after 1000 bootstrap runs. All sequences and accession numbers are shown in Supplementary Dataset 1, 2 and Table S1, respectively.

## 3. Results

### 3.1. Incubation with Natural Substrates Stimulated the Metamorphosis of A. serrata 

**Figure 1:**
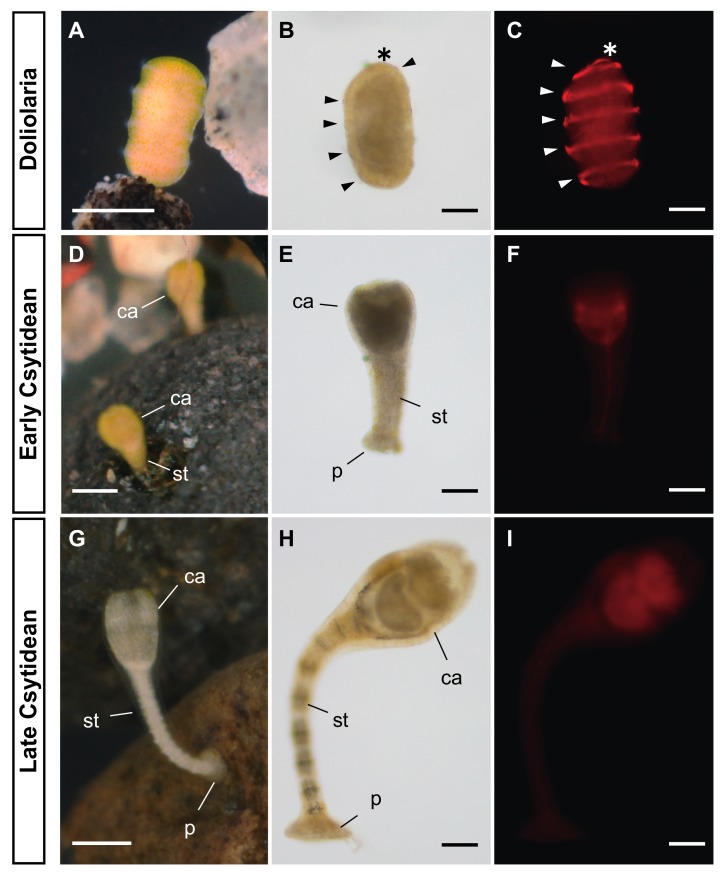
Development process and localization of ciliary bands in the feather star *A. serrata*. (**A**,**D**,**G**) show the living specimens of *A. serrata* larvae. Competent doliolaria larvae settle to the substrate with the apical tuft (**A**), then commence the metamorphosis process to transit to the csytidean larval phase (**D**, **G**; approximately two, four days after settlement, respectively). (**D**) shows the early cystidean larvae just after metamorphosis commenced. Calyx (ca), stalk (st) and adhesive plate (p) can be clearly observed in a few days after metamorphosis (**G**). (**B**,**C**,**E**,**F**,**H**,**I**) indicates the fixed embryos labeled with anti-acetylated tubulin antibody in doliolaria, early cystidean and late cystidean larvae, respectively (light field; **B**, **E** and **H**, observation of fluorescence; **C**, **F** and **I**). The specific fluorescence in ciliary bands (arrow heads) and apical tuft (asterisk) were observed in doliolaria larvae (**C**), whereas no specific fluorescence was observed in cystidean larvae (**F**,**I**). Scale bars: 250 µm (**A**,**D**,**G**), 100 µm (**B**,**C**,**E**,**F**,**H**,**I**).

We collected adult specimens of *A. serrata* with fertilized eggs or embryos in their pinnular surface from Misaki (Miura, Kanagawa Prefecture, Japan) and Onahama (Iwaki, Fukushima Prefecture, Japan). Doliolaria larvae hatch from the pinnular surface and swim in the water column using ciliary bands (Figure 1A). As described in the same genus species, *A. mediterranea* [28], doliolaria larvae of *A. serrata*, have five ciliary bands and an apical tuft that can be labeled by anti-acetylated tubulin antibody (Figure 1B,C). Within a few days after hatching, larval development reaches a plateau, and larvae become competent for metamorphosis. Then, doliolaria larvae attach to a substrate with adhesive tufts and transition to cystidean larvae through development of calyx and adhesive plates, the elongation of stalks and the disappearance of ciliary bands (Figure 1A–I). This process begins immediately after settlement, but it takes approximately two days for the stalk and other structures to be clearly observed. After metamorphosis completion, cystidean larvae transit to pentacrinoid larvae by the formation of tube feet, opening of the mouth and finally discard the stalk to become juveniles and start free-swimming life. 

Although the metamorphosis process of several species of crinoid is described in detail [28,29,30,31], how the larvae of crinoids determine the proper site for settlement is still debated. Previously, it was reported that the larvae of feather star aggregate and settle to the bottom of dishes in the laboratory [31]. On the other hand, just as larvae of several species of feather stars respond to natural substrates such as fragments of shell and coral [32], the reception of environmental cues would be required for their settlement. Here, we examined whether larvae of *A. serrata* can respond to environmental cues for metamorphosis by incubation of its doliolaria larvae with natural sands from the habitat of adult specimens (Miura, Kanagawa Prefecture, Japan).

**Figure 2:**
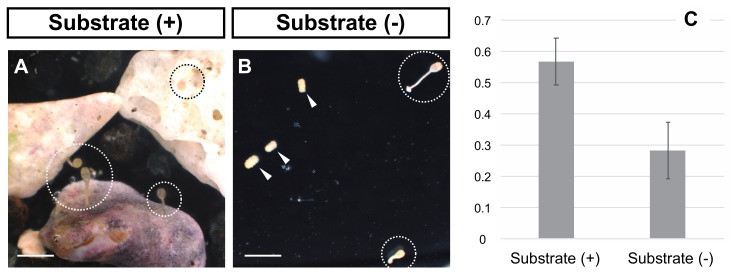
Effects of the presence or absence of substrates on settlement and metamorphosis. (**A**,**B**) indicate the doliolaria (arrowheads) or cystidean (dotted line circles) larvae incubated for six days with or without substrate, respectively. Scale bars: 1 mm. (**C**) shows the metamorphosis ratio for each treatment.

We reared competent doliolaria larvae of *A. serrata* with or without substrates for six days and counted the number of individuals that metamorphosed during this period. We found that approximately 30% of larvae metamorphosed to cystidean larvae in the absence of substrates (17 of 60 larvae from two batches, Figure 2A,C, Table S2). On the other hand, the number of metamorphosed larvae doubled in the presence of substrate (34 of 60 larvae from two batches, Figure 2B,C, Table S2). Larvae settled to the bottom of the plates or substrates and normally metamorphosed to cystidean larvae through the development of calyx, stalk and adhesive plates (Figure 2A,B). Significant differences in the metamorphosis ratios were observed between treatments (*P* = 0.0423, ANOVA). The response to the substrate did not differ between the Misaki and Onahama samples, as the metamorphosis ratio values were similar (Misaki: 13 of 20 larvae vs. Onahama: 21 of 40 larvae; Table S2), although a statistical analysis was not possible due to the small number of samples. These data suggest that the presence of environmental cues stimulated the commencement of metamorphosis.

### 3.2. Exogenous RA Treatment Induced the Metamorphosis of A. serrata

Next, we investigated the role of RA signaling in the metamorphosis of *A. serrata*. RA signaling plays a variety of developmental roles in chordates [33], through the synthesis of RA by RALDH (retinal dehydrogenase) and its binding to receptors such as RAR (retinoic acid receptor) and RXR (retinoid x receptor) to regulate downstream gene expression [33,34]. Although we could not conduct a genomic survey of *A. serrata* due to poor genomic information on the species, we identified the RA signaling components in transcriptome data from the sea lily *M. rotundus* (single genes: *raldh*, *rar*, and *rxr*; Figures S1 and S2), suggesting that the RA signaling machinery is conserved in the crinoid lineage.

**Figure 3:**
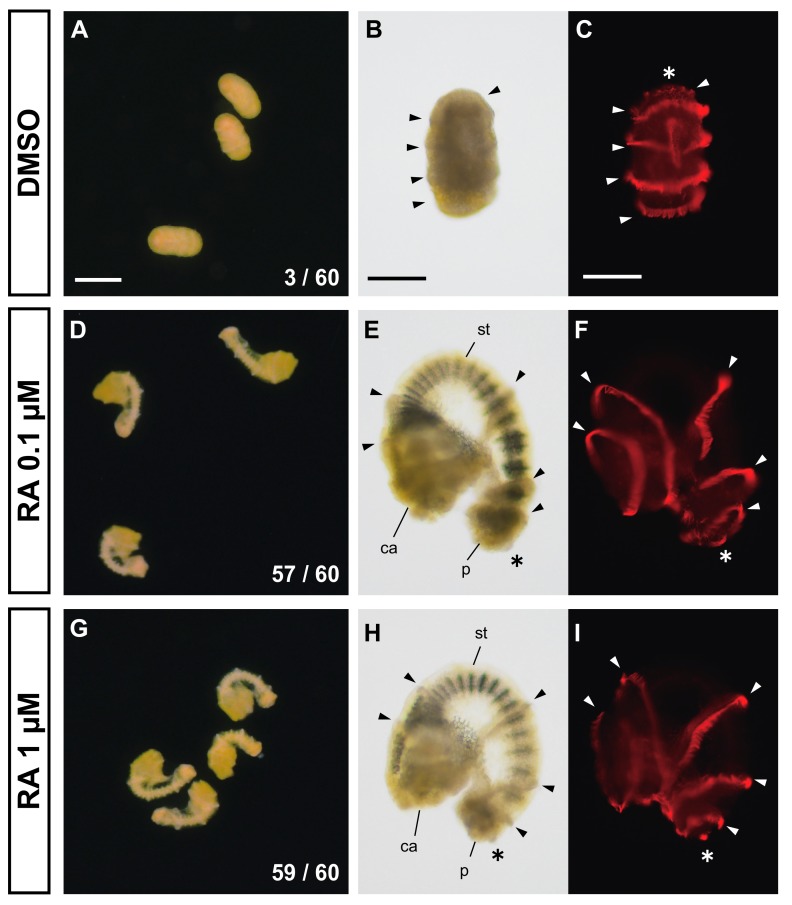
Induction of metamorphosis by exogenous RA treatment. (**A**–**I**) respectively show the larvae treated for 96 h with DMSO, RA 0.1 µM and RA 1 µM. While almost all doliolaria larvae did not metamorphose to cystidean larvae (**A**), metamorphosis was induced by the treatment of RA 0.1 µM and RA 1 µM (**D** and **G**, respectively). The numbers in (**A**,**D**,**G**) refer to “the number of metamorphosed larvae” / “the number of treated larvae”. (**B**,**C**,**E**,**F**,**H**,**I**) indicate the fixed larvae labeled with anti-acetylated tubulin antibody after DMSO, RA 0.1 µM and RA 1 µM treatment, respectively (light field; **B**, **E** and **H**, observation of fluorescence; **C**, **F** and **I**). In RA treatment, metamorphosis was induced as the calyx (ca), stalk (st) and adhesive plate (p) can be clearly observed, whereas ciliary bands (arrowheads) and apical tuft (asterisk) did not disappear (**E**,**F**,**H**,**I**) like in doliolaria larvae with DMSO treatment (**C**). Scale bars: 250 µm (**A**), 125 µm (**B**,**C**).

First, we treated competent doliolaria larvae of *A. serrata* for four days, with exogenous all-*trans* RA (0.1 or 1 µM) without substrates (Figure 1). We judged whether the larvae were metamorphosed by clear formation of the calyx, stalk and adhesive plate. In the control experiments (DMSO treatment), almost no larvae metamorphosed within four days after treatment (3 of 60 larvae from two batches, Figure 3A, Table S3), whereas exogenous RA treatments induced the metamorphosis process, including the development of calyx, stalk and adhesive plates (0.1, 1 µM; 57, 59 of 60 larvae from two batches, respectively; Figure 3D,G, Table S3). Metamorphosis was induced within 24 h after treatment and continued to proceed until 72–96 h after treatment so that the calyx and stalk were gradually more clearly observed (Figure S3). Spicules were observed in the calyx and stalk of individuals in which metamorphosis was induced (Figure 3D,E,G,H). The time scales of development and morphogenesis after the induction of metamorphosis by RA are similar to those of the transition of doliolaria larvae to cystidean larvae after settlement during normal development (Figure 1 and Figure 3). Therefore, the metamorphosis induced by RA without substrates was similar in structure and time scale to the metamorphosis in normal development, suggesting that RA is an endogenous regulator of metamorphosis.

However, no disappearance of the ciliary bands was observed in the metamorphosis induction by RA treatment (Figure 3E,F,H,I), although ciliary bands normally disappear during metamorphosis (Figure 1E,F,H,I). Our observation with immunofluorescence showed that the metamorphosis-induced larvae still have five ciliary bands and an apical tuft, like the control or normal development larvae (Figure 1 and Figure 3). These data suggest that the regulatory mechanism for the disappearance of the ciliary bands is independent of the formation of the calyx, stalk and adhesive plate. In other words, RA would not regulate the disappearance of the ciliary bands.

### 3.3. Endogenous RA Synthesis is Required for the Metamorphosis of A. serrata

To test whether the endogenous synthesis of RA is necessary for the metamorphosis process in *A. serrata*, we examined RALDH inhibitor (DEAB) treatment and its effects on metamorphosis. As shown above, natural sand from the habitat of adult specimens stimulated metamorphosis (Figure 2). Thus, we treated larvae with DEAB (300 µM) in seawater containing natural sand for six days and investigated its effect on settlement and metamorphosis for up to six days after treatment. We evaluated attachment of larvae to the external substrate by an adhesive tuft as settlement and judged whether the larvae were metamorphosed by clear formation of the calyx, stalk, and adhesive plate.

**Figure 4:**
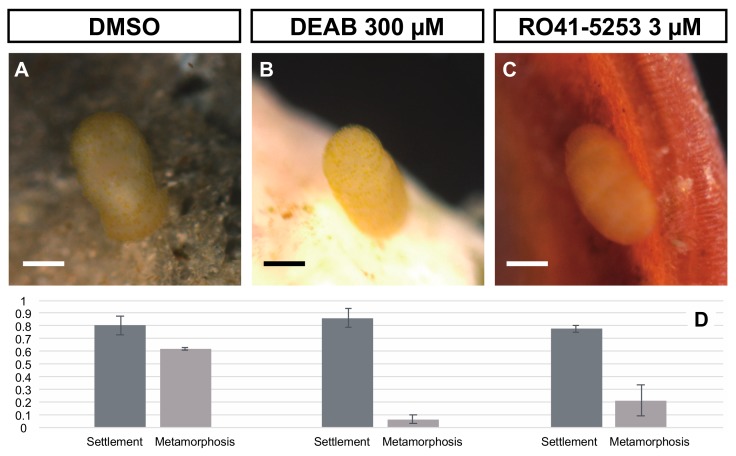
Effects of DEAB or RO treatment on settlement and metamorphosis. We treated doliolaria larvae with DMSO (control), DEAB 300 µM or RO 3 µM and examined the effects on settlement and metamorphosis. (**A**–**C**) show the settled larvae on substrates (natural sands from their adult habitat) in DMSO, DEAB and RO treatments. The ratio of settlement and metamorphosis is shown in (**D**) (dark gray; settlement ratio, light gray; metamorphosis ratio). Scale bars: 125 µm.

In both the control (DMSO) and DEAB 300 µM treatment, doliolaria larvae showed specific behaviors before metamorphosis, such as crawling around the substrate. Then, up to six days after treatment, we found that most of the larvae normally settled to substrates (DMSO; 29 of 36 larvae from two batches, DEAB; 31 of 36 larvae from two batches, Figure 4A,B,D, Table S4). We did not detect any significant differences in effect on the settlement between treatments (P = 0.45, ANOVA). However, although 62% of the larvae metamorphosed into cystidean larvae in the DMSO control (18 of 29 larvae from two batches, Figure 4D, Table S4), only a few larvae metamorphosed in the presence of DEAB treatment (2 of 31 larvae from two batches, Figure 4D, Table S4). DEAB inhibited metamorphosis significantly (*P* < 0.001, ANOVA). These data suggest that endogenous RA synthesis did not affect settlement but was required to commence metamorphosis.

### 3.4. RA Binding with RAR is Required for the Metamorphosis of A. serrata 

In a typical RA signaling pathway, the reception of RA by RAR has been shown to be essential for signal transduction [33,35]. Therefore, we examined whether the reception of RA by RAR is necessary to commence metamorphosis. During the above experiment, we also treated larvae for six days with the RARα antagonist, RO41-5253 (RO), which was used in the previous work with starfish [10]. As in the DMSO or DEAB treatment, larvae treated with RO 1 µM also showed specific behavior before metamorphosis, and most of them settled on the substrate (28 of 36 larvae from two batches, Figure 4C,D, Table S4). There was no statistically significant difference in the effects on settlements between RO treatment and control (*P* = 0.308, ANOVA). However, in the RO treatment, only a very small number of settled larvae were able to metamorphose (6 of 28 larvae from two batches, Figure 4C, D, Table S4). Significant differences in the metamorphosis ratios were observed between treatments (*P* < 0.001, ANOVA).

**Figure 5:**
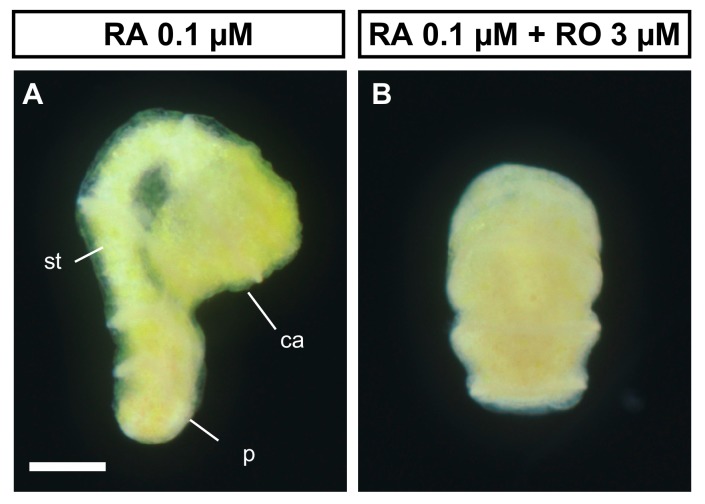
RO treatment suppressed the induction of metamorphosis by RA. RA 0.1 µM treatment with doliolaria larvae induced the metamorphosis (**A**), while this induction was suppressed by adding RO 3 µM (**B**). ca; calyx, st; stalk and p; adhesive plate. Scale bar: 125 µm.

We also investigated whether RO treatment suppressed the induction of metamorphosis by RA treatment. As shown above, we found that treatment with 0.1 µM exogenous RA induced the metamorphosis of doliolaria larvae 72 h after treatment (16 of 16 larvae from two batches, Figure 5A, Table S5). Conversely, treatment of larvae with 0.1 μM RA plus 1 μM RO did not induce metamorphosis in most larvae (2 of 16 larvae from two batches, Figure 5B, Table S5). Although a statistical analysis was not possible due to the small number of samples in this experiment, the presence of RO suppressed metamorphosis. The effect of exogenous RA treatment on metamorphosis was examined 96 h after the treatment (Figure 3). Although the effects of exogenous RA, DEAB and RO treatments on metamorphosis were examined up to 96 h after the treatment, the RA + RO treatment had a fatal effect on larvae at 96 h after treatment, as the larval body swelled. Thus, the effect on metamorphosis was examined 72 h after RA + RO treatment. Nonetheless, our experimental data suggest that RA binding to RAR is required for the metamorphosis of *A. serrata*.

## 4. Discussion

### 4.1. Metamorphosis Regulation by RA Signaling in the Ancestor of Living Echinoderms

**Figure 6:**
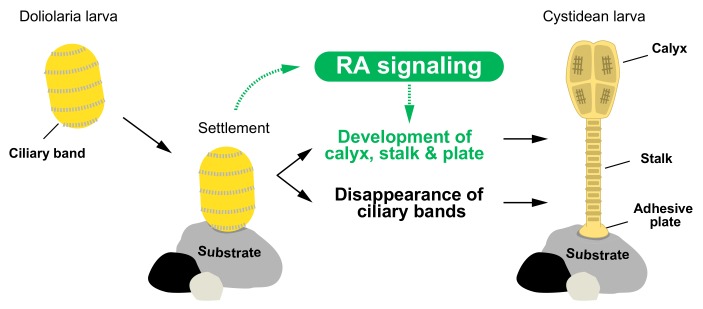
Hypothetical regulatory mechanism for metamorphosis of feather stars. Black arrows indicate the developmental process of *A. serrata*. Competent doliolaria larvae settle to external substrate with reception of environmental cues to commence metamorphosis process. Finally, transition to sessile cystidean larvae is completed through the disappearance of ciliary bands and the development of calyx, stalk and adhesive plate. We hypothesized that RA signaling mediates the metamorphosis process such as the development of calyx upon the reception of environmental signals (shown in green).

In this study, we hypothesized that RA signaling mediates the metamorphosis process, including development of stalk and calyx, once environmental cues are received in feather stars (Figure 6). Although our idea is supported by interfering with RA signaling at the levels of RA synthesis and RAR-activation [33], we recognize that our conclusion will become more robust after future studies, including testing if all trans-retinaldehyde, the RA precursor molecule, or other forms of RA, are able to promote metamorphosis. We also should determine if RA signaling is activated after settlement through a quantitative polymerase chain reaction analysis of downstream genes. 

In addition, the disappearance of ciliary bands was independent of RA signaling, as the ciliary bands did not disappear in larvae in which metamorphosis was induced by exogenous RA treatment (Figure 3). Therefore, other regulatory components must be investigated to understand the comprehensive regulatory mechanism of the metamorphosis of feather stars. 

Our findings support that metamorphosis was RA-dependent in the ancestors of extant echinoderms. Crinoids (feather star and stalked sea lily) are the most basal group of extant echinoderms, forming a sister group with Eleutherozoa including other echinoderm taxa [13]. Both the feather star and stalked sea lily develop doliolaria-type larvae before settlement [36,37], although it should be noted that the stalked sea lily develops semidoliolaria stages but not a full doliolaria stage [36]. Thus, it is hypothesized that the ancestors of crinoids had a life cycle in which the doliolaria-type larvae metamorphosed into the cystidean larvae [37]. Namely, as shown in feather stars, it is suggested that metamorphosis is regulated by RA in the ancestor of crinoids. In addition, among the lineages of Eleutherozoa, we previously reported that the metamorphosis of starfish is regulated by RA signaling [10]. In both feather stars and starfish, RA signaling mediates the process of metamorphosis after receiving an environmental signal at settlement, suggesting that the developmental role of RA signaling is evolutionarily conserved. These findings support an ancient origin of RA-dependent metamorphosis during echinoderm evolution.

Although echinoderms have evolved various larval morphologies in each lineage [16], the metamorphosis regulatory mechanisms might be evolutionarily conserved, as in feather star and starfish. In this context, we should especially focus on metamorphosis regulation in sea urchins, which acquired larval skeletons and evolved a pluteus larval form [16]. The metamorphosis regulation in the sea urchin has been clarified in comparatively high detail [18,19]. Generally, thyroid hormone and histamine signaling modulate larval growth and competency acquisition, and nitric oxide signaling negatively controls the postsettlement process [18,19]. Despite the above findings, it has not been reported that RA signaling is involved in the metamorphosis regulation of sea urchin. In parallel with the investigations of metamorphosis regulaton, genomic survey revealed that the typical RALDH (Aldh1a family) genes are absent in the genome of sea urchin [38].

Note that the above information does not necessarily indicate that the metamorphosis of sea urchin is independent of RA signaling. Rather, RA signaling is expected to be functional even in sea urchin because other RA signaling components such as RAR and RXR were identified [38]. Furthermore, Aldh8 gene, which has the potential to synthesize RA, was also found in the genomic data of sea urchin, suggesting that RA signaling works in sea urchin without typical RALDH genes [39]. Therefore, to deepen our understanding of the evolution of metamorphosis regulation in echinoderms, we suggest that it is important to investigate the role of RA signaling in the metamorphosis of sea urchins. 

Finally, it would be interesting to know if RA signaling regulates the metamorphosis in sea cucumbers, which show gradual metamorphosis and a secondary bilateral axis [40], as well as in sea urchins. By studying the function of RA signaling in various echinoderms, we can better understand the evolution of the echinoderm life cycle.

### 4.2. Life Cycle Evolution from the Viewpoint of RA Signaling

The evolution of planktonic larvae in marine invertebrates has attracted great interest from many zoologists [1,3]. It has been hypothesized that the common ancestor of cnidarians and bilaterians had planktonic larvae based on the formation mechanism of an apical organ and body patterning [4,5,6,7]. Furthermore, endogenous RA is reported to mediate strobilation and metamorphosis in jellyfish and starfish, respectively, once environmental cues are received [9,10]. Our study also suggests that metamorphosis is regulated by RA signaling in echinoderm ancestors. Based on these findings, we hypothesized that RA has the function of transiting the life cycle in the common ancestor of cnidarians and bilaterians, suggesting that such functions have been co-opted to regulate strobilation and metamorphosis in cnidarians and echinoderms, respectively.

Further studies are required to reveal which processes RA regulates in the life cycle of the common ancestor. Although the life cycle evolution of cnidarians remains controversial, recent molecular phylogenic analyses support the polyp-first hypothesis, suggesting that the jellyfish stage is a derived feature in the lineage of cnidarians [41]. Thus, it is important to learn the ancestral function of RA signaling in cnidarians. In particular, it is of interest to investigate whether RA regulates the transition process of planktonic planula larva to sessile polyps in the ancestor of cnidarians, as in echinoderms. Previous studies with exogenous RA treatment provided insights into such functions. For example, Pennati et al. [42] examined RA treatment in the planula larvae of the hydrozoan *Clava multicornis* and reported influence on the anterior-posterior positioning of peptidergic neurons but not on the induction of polyp. Nevertheless, the RA signaling machinery is lacking in the anthozoan and several lineages of hydrozoans, as no RXR genes have been identified in their genomic data [9]. Because it is unclear whether *C. multicornis* has the RXR gene due to limited genetic information, it is difficult to reveal the ancestral function of RA in cnidarians through investigations without genomic surveys. We suggest that future studies should re-examine the function of RA with species with the RXR gene.

### 4.3. Insight into the Ancestral Function of RA Signaling

Although our data illuminate the ancestral function of RA signaling in echinoderms as a regulator of life cycle transition, its validity throughout the animal kingdom still requires further assessments. In particular, the following two points should be noted. The first is a study by Handberg-Thorsager et al. [12] using the marine annelid *Platynereis dumerilii*. This study clarified the detailed biochemical features of RA signaling and its developmental role in neurogenesis in *P. dumerilii*, suggesting that RAR ancestrally functions as a low-affinity sensor triggering neural differentiation [12]. This work reported no function in life cycle transition, although such a function might not be captured in their framework, which focused on the neurogenesis of embryos and the early nectochaete larval stage [12]. Namely, in *P. dumerilii*, it is reported that the late nectochaete larvae settle on external substrates and commence “settlement metamorphosis” to transition to the errant juvenile stage [43]. Therefore, we suggest that future studies should focus on the function of RA signaling in later stages, such as the late nectochaete larval stage or phase after settlement.

Second, in invertebrates of deuterostomes other than echinoderms, the regulation of metamorphosis by RA has not been reported. In particular, ascidians have a life cycle similar to that of many marine invertebrates, in which swimming larvae settle to the bottom and begin sessile life [44]. Furthermore, their metamorphosis regulatory mechanism has been clarified in detail [45], although there are no reports that RA signaling functions as a regulator of metamorphosis control. Instead, it has been suggested that RA signaling functions conservatively with other chordates, such as in the regulation of Hox gene expression [34]. In this context, it is important to determine whether metamorphosis was regulated by RA in the ancestor of the deuterostomes. In particular, we should investigate the role of RA signaling in hemichordates, a sister group of echinoderms. Although the life cycle of hemichordates is similar to that of echinoderms, where planktonic tornaria larvae metamorphose to juveniles after settlement [46], it is unclear whether their metamorphosis is regulated by RA signaling.

As described above, our study showed that we can approach the origin of larvae and the life cycle evolution from the viewpoint of life cycle regulation. Further research on various animal groups should lead to a comprehensive understanding of life cycle evolution.

## 5. Conclusions

We found that RA signaling mediates the metamorphosis of feather stars upon reception of environmental cues, suggesting that metamorphosis is regulated by RA signaling in echinoderm ancestors. As conclusion, our findings support the idea that RA has a function of transiting the life cycle in the common ancestor of cnidarians and bilaterians, and such functions have been co-opted to regulate strobilation and metamorphosis in cnidarians and echinoderms, respectively.

## Supplementary Materials

The following are available online at https://www.mdpi.com/2218-273X/10/1/37/s1, Figure S1: The phylogenic tree of ALDH gene family. Figure S2: The phylogenic tree of RAR, RXR and THR (Thyroid hormone receptor). Figure S3: Temporal change of larvae in metamorphosis induction by exogenous RA treatment. Table S1: The accession numbers of sequences for phylogenetic analysis. Table S2: Number of metamorphosed/treated larvae of each batch in substrate treatment experiment. Table S3: Number of metamorphosed/treated larvae of each batch in RA treatment experiment. Table S4: Number of settled or metamorphosed/treated larvae of each batch in DEAB or RO treatment experiment. Table S5: Number of metamorphosed/treated larvae of each batch in RA+RO treatment experiment. Supplementary dataset 1: The sequences for phylogenetic analysis of Raldh. Supplementary dataset 2: The sequences for phylogenetic analysis of RAR and RXR.
