# Supplementary material for: Retinoic Acid Signaling Regulates the Metamorphosis of Feather Stars (Crinoidea, Echinodermata): Insight into the Evolution of the Animal Life Cycle
Source: Biomolecules. 2019 Dec 25;10(1):37. doi: 10.3390/biom10010037 (PMC7023313; doi:10.3390/biom10010037)
Supplement: Supplementary file 1 [file biomolecules-10-00037-s001.zip › Supplementary files/Table S1.pdf]

Table S1

Species	gene	accession number	gene	accession number
Hs; *Homo sapiens*	*aldh1a1*	P00352.2	*thra*	P10827
	aldh1a2	O94788	*thrb*	P10828
	aldh1a3	P47895	*rara*	P10276
	aldh1b1	P30837	*rarb*	P10826
	aldh2	P05091	*rarg*	P13631
	aldh3a1	P30838	*rxra*	P19793
	aldh3a2	P51648	*rxrb*	P28702
	aldh3b1	P43353	*rxrg*	P48443
	aldh3b2	P48448		
	aldh4a1	P30038		
	aldh5a1	P51649		
	aldh6a1	Q02252		
	aldh7a1	P49419		
	aldh8a1	Q9H2A2		
	aldh9a1	P49189		
Mm; *Mus musculus*			*thra*	P63058
			thrb	P37242
			rara	P11416
			rarb	P22605
			rarg	P18911
			rxra	P28700
			rxrb	P28704
			rxrg	P37238
Xt; *Xenopus tropicalis*	*aldh1a1*	Q4VBE1		
	aldh1a2	Q9DEX5		
	aldh1a3	F7BV06		
	aldh1b1	F7DQF8		
	aldh2	Q6DJ49		
	aldh3a2	B1WBI3		
	aldh3b2	F6X8Y6		
	aldh4a1	A4QNJ0		
	aldh5a1	F6QFQ2		
	aldh6a1	F6SRL8		

	aldh7a1	F7BQF6		
	aldh8a1	F6UH88		
	aldh9a1	F6VC33		
Dr; *Danio rerio*	*aldh1a2*	Q90XS8	*thraa*	Q98867
	aldh1a3	Q0H2G3	*thrab*	U3JAT9
	aldh2a	Q8QGQ2	*thrb*	Q9PVE4
	aldh2b	Q6TH48	*raraa*	Q90271
	aldh3a1	X1WBM4	*rarab*	Q7ZTI3
	aldh3a2a	A0A2R8PW97		
	aldh3a2b	E9QH31	*rarga*	Q91392
	aldh3b1	Q90ZZ7	*rargb*	A2T928
	aldh4a1	Q7SY23		
	aldh5a1	A0A0R4IIB7		
	aldh6a1	Q6DHT4		
	aldh7a1	Q803R9		
	aldh8a1	Q66I21		
	aldh9a1a1	Q7ZVB2		
	aldh9a1a2	B0S7W5		
	aldh9a1b	Q802W2		
Bf; *Branchiostoma floridae*	*aldh1a_1*	C3ZGK4	*rxr*	Q8MX78
	aldh1a_2	C3ZG63		
Bl; *Branchiostoma lanceolatum*			*rar*	O18608
Ci; *Ciona intestinalis*	*aldh1a_1*	A0A1W2WB51	*rar*	Q4H2W1
	aldh1a_2	A0A1W5BCT1	*rxr*	Q4H2U9
	aldh1a_3	A0A1W2WDC1		
	aldh2	A0A1W5B7N8		
Pm; *Polyandrocarpa misakiensis*			*rxr*	K7ZLP3
Sk; *Saccoglossus kowalevskii*	*aldh1a_1*	XP_006823779.1	*rar*	XP_002742241.1
	aldh1a_2	XP_006822197.1	*rxr*	D2XNK4
	aldh1a_3	XP_002736989.1		
	aldh1a_4	XP_002731204.1		
	aldh1a_5	XP_006824634.1		
	aldh2	XP_006816163.1		
Sp; *Strongylocentrotus purpuratus*	*aldh2_1*	SPU_007284	*thr*	SPU_025239
	aldh2_2	SPU_023801	*rar*	SPU_016523
	aldh5a1_1	SPU_007492.1	*rxr*	SPU_028422

	aldh5a1_2	SPU_016767.1		
	aldh6a1	SPU_026493.1		
	aldh7	SPU_024895.3a		
	aldh8a1_1	SPU_017403.1		
	aldh8a_2	SPU_000522.1		
	aldh9	SPU_002901.3a		
Pp; *Patiria pectinifera*	*raldha*	LC379260	*rar*	LC379258
	raldhb	LC379261	*rxr*	LC379259
	raldhc	LC379262	*thr*	*
	aldh2	*		
Dm; *Drosophila melanogaster*			*usp*	P20153
Rc; *Reishia clavigera*			*rar*	T2HRZ4
			rxr	E9RHD8
Ls; *Lymnaea stagnalis*			*rar*	D5LIR6
			rxr	Q5I7G2
Tc; *Tripedalia cystophora*			*rxr*	O96562

* Sequences were not deposited to databank but available from supplementary dataset 1 or 2 of this work.
