# Supplementary material for: Retinoic Acid Signaling Regulates the Metamorphosis of Feather Stars (Crinoidea, Echinodermata): Insight into the Evolution of the Animal Life Cycle
Source: Biomolecules. 2019 Dec 25;10(1):37. doi: 10.3390/biom10010037 (PMC7023313; doi:10.3390/biom10010037)
Supplement: Supplementary file 1 [file biomolecules-10-00037-s001.zip › Supplementary files/Table S2.pdf]

Table S2

| | Substrate (+) | | Substrate (-) | |
|---|---|---|---|---|
| | number | metamorphosis | number | metamorphosis |
| batch 1-1 | 10 | 8 | 10 | 1 |
| batch 1-2 | 10 | 5 | 10 | 0 |
| batch 2-1 | 10 | 8 | 10 | 2 |
| batch 2-2 | 10 | 4 | 10 | 4 |
| batch 2-3 | 10 | 4 | 10 | 4 |
| batch 2-4 | 10 | 5 | 10 | 6 |
| Total | 60 | 34 | 60 | 17 |
