# Supplementary material for: Retinoic Acid Signaling Regulates the Metamorphosis of Feather Stars (Crinoidea, Echinodermata): Insight into the Evolution of the Animal Life Cycle
Source: Biomolecules. 2019 Dec 25;10(1):37. doi: 10.3390/biom10010037 (PMC7023313; doi:10.3390/biom10010037)
Supplement: Supplementary file 1 [file biomolecules-10-00037-s001.zip › Supplementary files/Table S3.pdf]

Table S3

	DMSO		RA 0.1 μM		RA 1 μM	
	number	metamorphosis	number	metamorphosis	number	metamorphosis
batch 1	20	2	20	20	20	20
batch 2-1	10	0	10	9	10	10
batch 2-2	10	0	10	8	10	10
batch 2-3	10	0	10	10	10	9
batch 2-4	10	1	10	10	10	10
Total	60	3	60	57	60	59
