# Supplementary material for: Retinoic Acid Signaling Regulates the Metamorphosis of Feather Stars (Crinoidea, Echinodermata): Insight into the Evolution of the Animal Life Cycle
Source: Biomolecules. 2019 Dec 25;10(1):37. doi: 10.3390/biom10010037 (PMC7023313; doi:10.3390/biom10010037)
Supplement: Supplementary file 1 [file biomolecules-10-00037-s001.zip › Supplementary files/Table S4.pdf]

Table S4

	DMSO			DEAB			RO		
	treatment	settlement	metamorphosis	treatment	settlement	metamorphosis	treatment	settlement	metamorphosis
Batch 1	12	8	5	12	12	1	12	9	2
Batch 2-1	12	11	7	12	9	1	12	10	4
Batch 2-2	12	10	6	12	10	0	12	9	0
Total	36	29	18	36	31	2	36	28	6
