# Supplementary material for: Retinoic Acid Signaling Regulates the Metamorphosis of Feather Stars (Crinoidea, Echinodermata): Insight into the Evolution of the Animal Life Cycle
Source: Biomolecules. 2019 Dec 25;10(1):37. doi: 10.3390/biom10010037 (PMC7023313; doi:10.3390/biom10010037)
Supplement: Supplementary file 1 [file biomolecules-10-00037-s001.zip › Supplementary files/Table S5.pdf]

Table S5

	RA 0.1 μM		RA 0.1 μM + RO 3 μM	
	number	metamorphosis	number	metamorphosis
batch 1	10	10	10	0
batch 2	6	6	6	2
Total	16	16	16	2
