# Supplementary figures and images for: Retinoic Acid Signaling Regulates the Metamorphosis of Feather Stars (Crinoidea, Echinodermata): Insight into the Evolution of the Animal Life Cycle
Source: Biomolecules. 2019 Dec 25;10(1):37. doi: 10.3390/biom10010037 (PMC7023313; doi:10.3390/biom10010037)

Figure S1

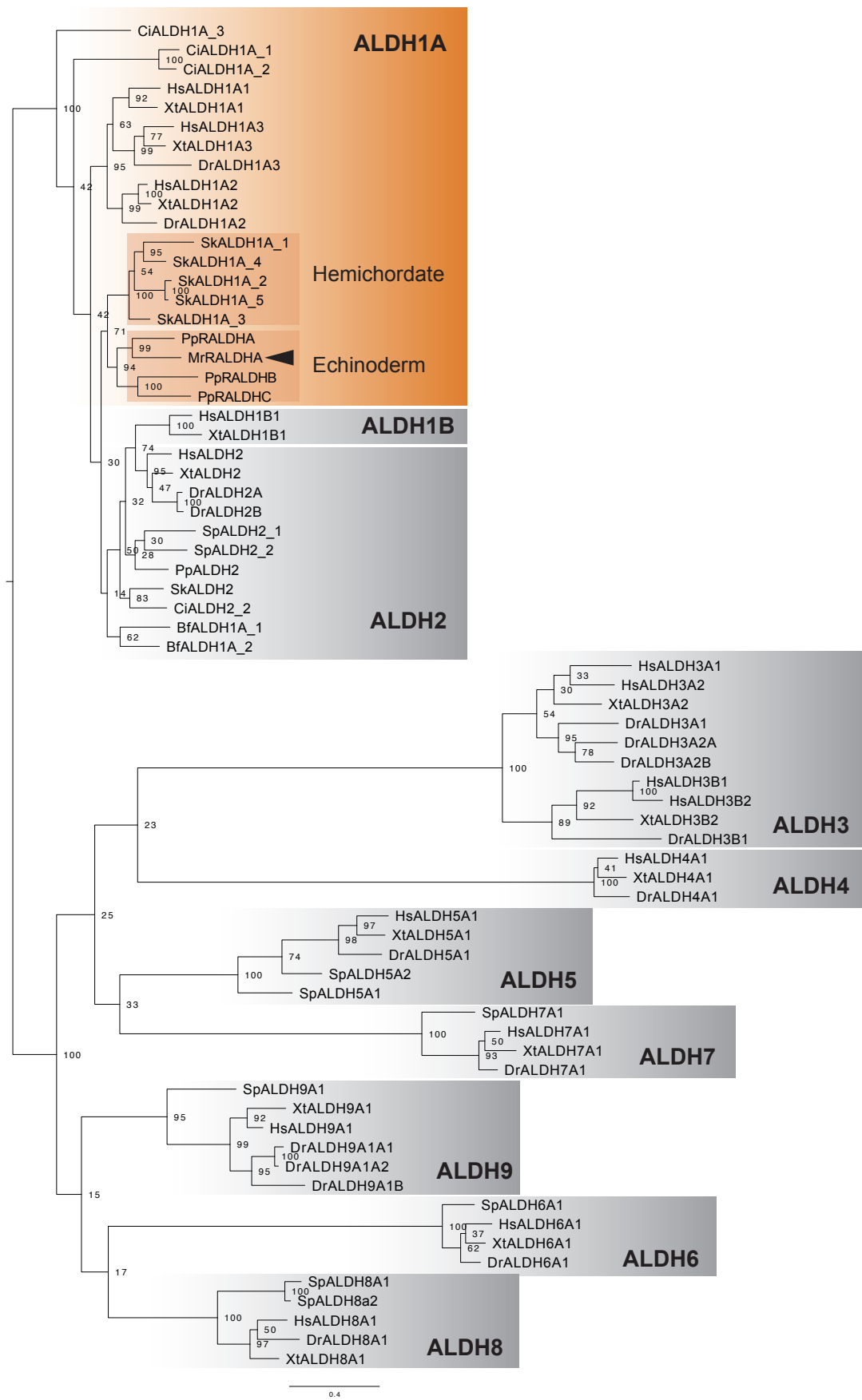

Supplement: Supplementary file 1 [file biomolecules-10-00037-s001.zip › Supplementary files/Figure S1.pdf]

Figure S2

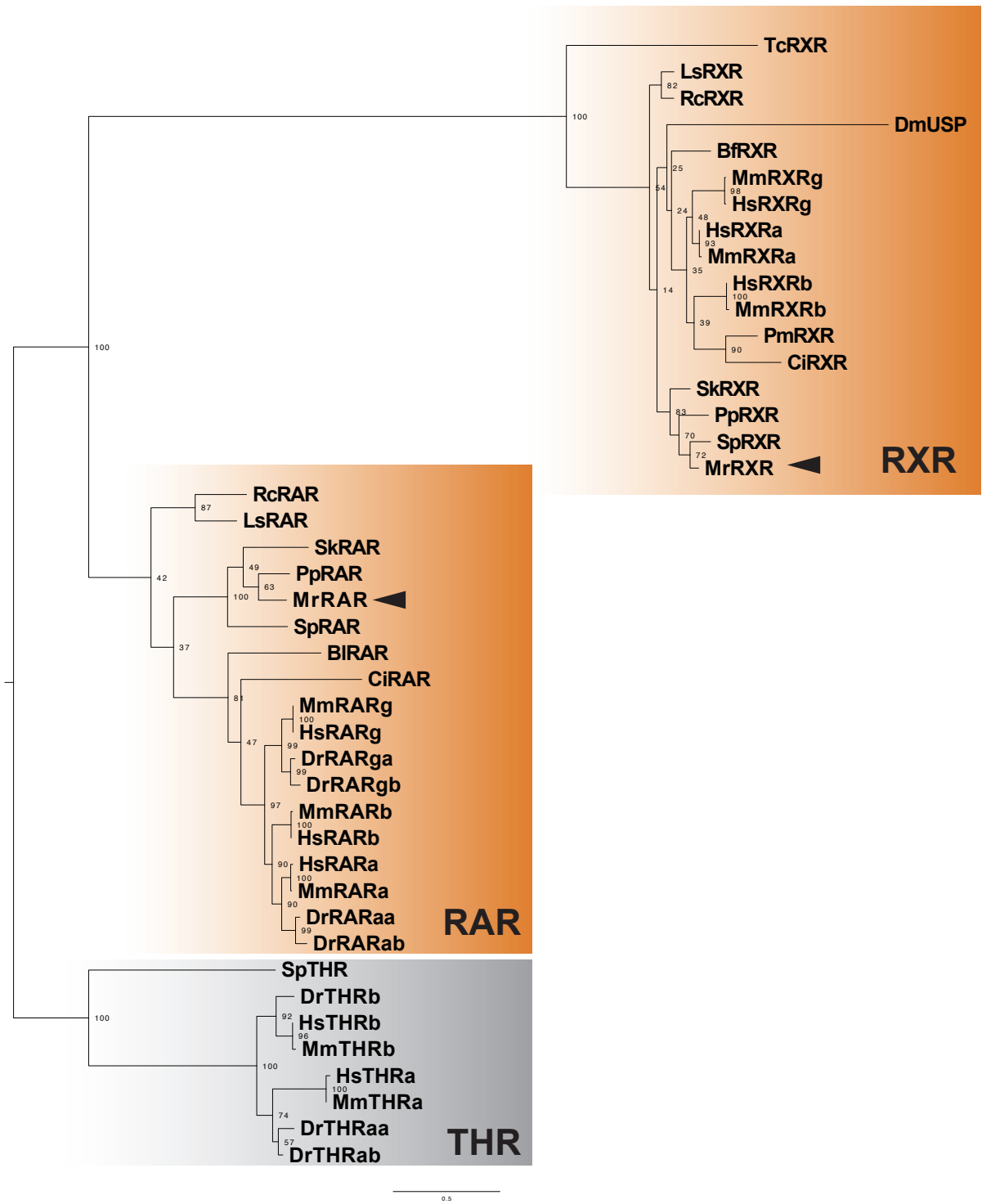

Supplement: Supplementary file 1 [file biomolecules-10-00037-s001.zip › Supplementary files/Figure S2.pdf]

Figure S1

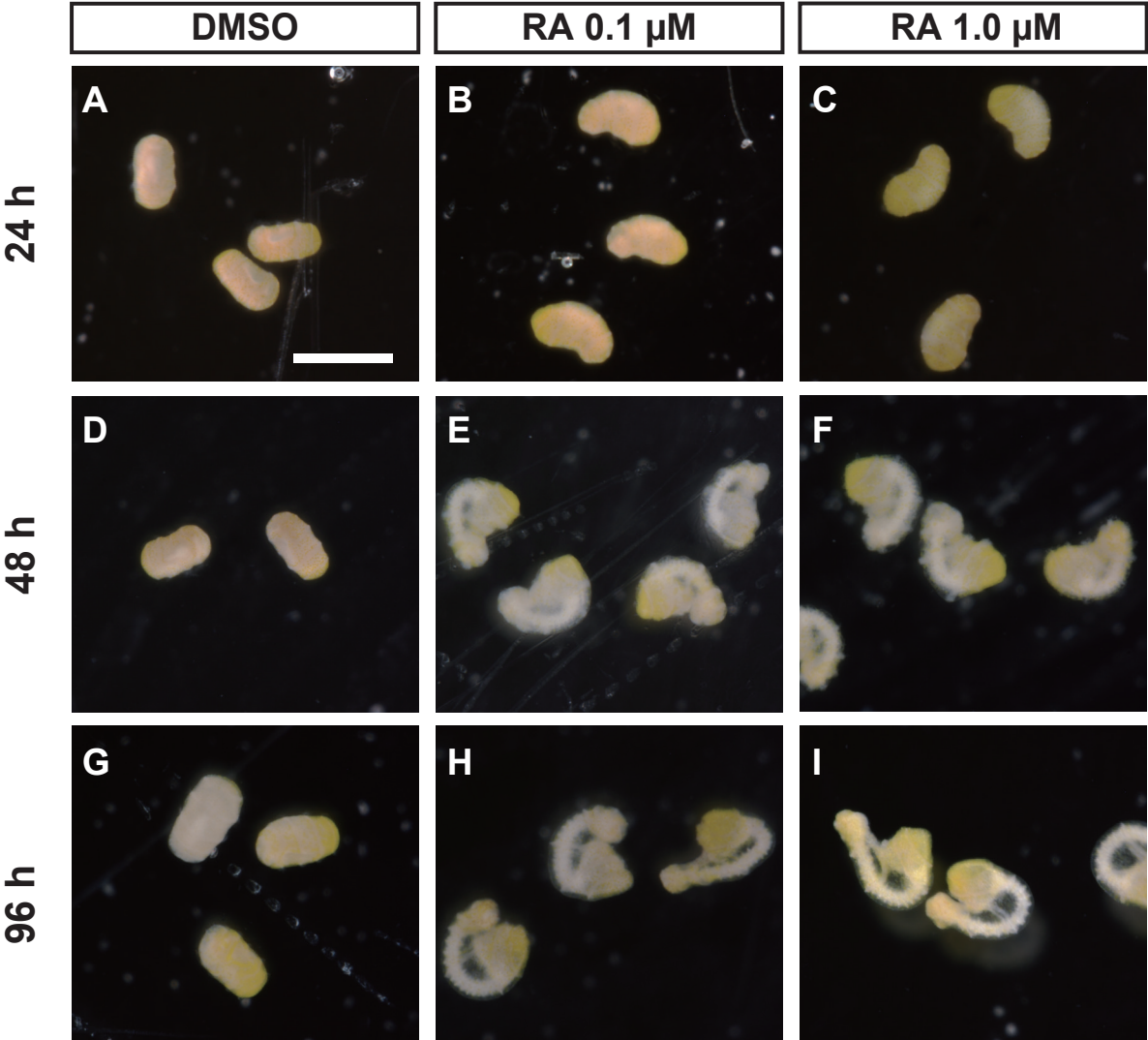

Supplement: Supplementary file 1 [file biomolecules-10-00037-s001.zip › Supplementary files/Figure S3.pdf]
